# Initial Exposure of Wax Foundation to Agrochemicals Causes Negligible Effects on the Growth and Winter Survival of Incipient Honey Bee (*Apis mellifera*) Colonies

**DOI:** 10.3390/insects10010019

**Published:** 2019-01-08

**Authors:** Alexandria N. Payne, Elizabeth M. Walsh, Juliana Rangel

**Affiliations:** Department of Entomology, Texas A&M University, 2475 TAMU, College Station, TX 77843-2475, USA; arekkusu@tamu.edu (A.N.P.); walshe@tamu.edu (E.M.W.)

**Keywords:** agrochemicals, *Apis mellifera*, honey bees, beeswax, colony growth, pesticides, toxicology, miticides

## Abstract

Widespread use of agrochemicals in the U.S. has led to nearly universal contamination of beeswax in honey bee hives. The most commonly found agrochemicals in wax include beekeeper-applied miticides containing *tau*-fluvalinate, coumaphos, or amitraz, and field-applied pesticides containing chlorothalonil or chlorpyrifos. Wax contaminated with these pesticides negatively affects the reproductive quality of queens and drones. However, the synergistic effects of these pesticides on the growth and survival of incipient colonies remain understudied. We established new colonies using frames with wax foundation that was pesticide free or contaminated with field-relevant concentrations of amitraz alone, a combination of *tau*-fluvalinate and coumaphos, or a combination of chlorothalonil and chlorpyrifos. Colony growth was assessed by estimating comb and brood production, food storage, and adult bee population during a colony’s first season. We also measured colony overwintering survival. We found no significant differences in colony growth or survivorship between colonies established on pesticide-free vs. pesticide-laden wax foundation. However, colonies that had *Varroa destructor* levels above 3% in the fall were more likely to die over winter than those with levels below this threshold, indicating that high *Varroa* infestation in the fall played a more important role than initial pesticide exposure of wax foundation in the winter survival of newly established colonies.

## 1. Introduction

Honey bees (*Apis mellifera*) provide over $15 billion annually to the U.S. economy by pollinating approximately 35% of commercially grown crops that depend directly on their pollination services to produce economically viable yields [1]. Because of populous colonies and ease of transportation, honey bees are preferred among other insect species for commercial crop pollination [2,3,4,5]. But despite their importance to our agroecosystems, managed honey bee populations continue to decline due to stresses mainly caused by poor nutrition, pests and pathogens, queen failure, and pesticide exposure [6,7].

In 2012, approximately 915 million acres of commercial cropland and 88 million households in the U.S. were treated with pesticides [8]. Despite their pervasive presence in the environment, pesticide risk assessments for insect pollinators are primarily based on a pesticide’s acute lethality to honey bees and thus present an inaccurate picture of pollinator–pesticide interactions. These assessments do not take into account the effects of pesticides on native and non-honey bee pollinators, and they do not consider sub-lethal effects or synergistic and multi-modal routes of exposure to honey bees when a pesticide’s registration or its renewal is processed. Due to the close association between commercial crops and honey bees, as well as high pesticide use in agricultural croplands, the effects of pesticide exposure on honey bee health are legitimate causes for active research [9,10].

Problematic pesticides for honey bee health can generally be split into two categories: those that are farmer applied to control agricultural pests, which are inadvertently brought into the hive by exposed foragers, or those that are intentionally introduced to colonies by beekeepers to control the ectoparasitic mite *Varroa destructor*. The miticides most widely used for *Varroa* control in the last three decades have had as active ingredients the pyrethroid *tau*-fluvalinate (active ingredient in Apistan^®^), the organophosphate coumaphos (active ingredient in Checkmite+^®^), or the formamidine amitraz (active ingredient in Apivar^®^). Studies have shown that the majority of honey bee colonies in the U.S. harbor beeswax containing alarming levels of these miticides [11,12]. Mullin et al. [12] sampled colonies from commercial beekeeping operations across the U.S. and found 87 different pesticides and their metabolites in 259 wax samples analyzed. The most contaminated wax sample contained 39 pesticides and their metabolites. On average, each wax sample contained residues from eight pesticides, with half (49.9%) of all samples containing one or more systemic insecticides. In particular, even though the miticides *tau*-fluvalinate and coumaphos are practically not used anymore for *Varroa* control in most of the U.S. [12], they were pervasive in wax and were found together in 77.7% of all the bee, pollen, and wax samples analyzed. Therefore, we decided to test the effects on colony growth of exposure of the wax comb to these two miticides combined into one treatment. Amitraz and its metabolites, 2,4-dimethylphenylformamide (DMPF) and 2,4-dimethyl aniline (DMA), was the third most commonly found miticides found in wax, so we decided to test the effects of wax exposure to amitraz (alone) on colony growth. The list of the five most ubiquitous pesticides found in wax also contained the fungicide chlorothalonil and the organophosphate chlorpyrifos as the two most commonly found agrochemicals. We therefore decided to test the effects on colony growth of comb exposure to a combination of these two non-miticide products. The high prevalence of *tau*-fluvalinate, coumaphos, amitraz, chlorothalonil, and chlorpyrifos has been confirmed in more recent years by another study conducted with three migratory beekeeping operations in the Eastern U.S. [11].

Previous studies have looked at the effects of acute doses of pesticides on immature and adult honey bees at the individual level. Some of these studies are controversial, however, because they have examined bees exposed to unrealistically high pesticide dosages—sometimes multiple folds higher than pesticide exposure found in the field [13,14,15]. Furthermore, very few of these studies have looked at the overall effects of pesticide exposure at the colony level. More recently, researchers have attempted to measure the “exposome”, or a colony’s total exposure to pesticides, through routes that vary depending on the environment in which colonies are managed [10,11].

In this study, we explored whether pesticide residues in wax at field-relevant concentrations affect the growth and survival of newly established colonies, using the highest concentrations of these pesticides in wax samples found in the field [12]. We did so by coating frames of plastic foundation with pesticide-free beeswax and then spraying them with a pesticide-free diluent, or with diluent containing field-relevant doses of amitraz, *tau*-fluvalinate and coumaphos, or chlorothalonil and chlorpyrifos. We compared the amount of comb built, brood produced, and food stored, as well as the adult population and seasonal colony survival of colonies established on pesticide-free wax foundation compared to those established on pesticide-laden wax foundation. Determining whether pesticide residues in wax foundation affect the health of incipient colonies will provide valuable information to beekeepers in order for them to better manage healthier and more productive hives.

## 2. Materials and Methods

### 2.1. Study Site and Colony Establishment

This study was conducted at an apiary located in Bryan, TX, USA (30.66290012 N, −96.47334016 E) from May 2017 to May 2018. All colonies were established following a previously described protocol [16,17]. A total of 30 packages consisting of 3 lbs of worker bees, or approximately 10,476 individuals [18], were created from larger, unrelated source colonies provided by ETzzzBzzz Apiary of College Station, TX, USA. Each package of bees was shaken into a standard package box (15 cm × 25 cm × 35 cm), as described by Seeley and Visscher [19], along with a mated and caged Italian queen. All queens were received in May 2017 from Olivarez Honey Bees, Inc. (Orland, CA, USA). The packages were fed ad libitum with a 50:50 *w*/*v* sucrose solution, allowing the bees to reach the natural inclination of wax production for new colony establishment [20]. Three days later, each package was installed by shaking the bees into a five-frame “nucleus” hive along with the caged queen. This was done on 18 May 2017, which marked the colony establishment date and the first day of the study. Each nucleus hive was comprised of five frames of alternating full or partial Plasticell foundation (Pierco Beekeeping Equipment^®^, Riverside, CA, USA) coated with a layer of molten beeswax (see Section 2.2 below). Frames with full foundation allowed bees to construct only worker comb (and consequently the production of only worker brood), while frames with partial foundation allowed bees to build either worker or drone comb depending on the colony’s needs. The caged queens were released from their cages two days after establishing the hives once it was evident they had been accepted by their workers.

### 2.2. Pesticide Treatment of Wax Foundation

Each Plasticell foundation frame was coated with a layer of pesticide-free, cosmetic grade beeswax pellets (Koster Keunen Inc., Watertown, CT, USA) previously melted in a water bath. Once the molten wax layer had dried, a frame was sprayed using separate all-purpose sprayers containing either acetone only (control group) or acetone mixed with pesticides at the high concentrations found in wax samples by Mullin et al. [12]. Because we used the highest concentrations previously found in wax samples [12], our study represents a “worst case” scenario of pesticide exposure of beeswax foundation and does not account for other routes of additional pesticide exposure. Frames in the treatment groups were sprayed with either (1) 4.3 mg amitraz (“amitraz” group), (2) a mix of 20.4 mg fluvalinate and 9.2 mg coumaphos (“F+C” group), or (3) a mix of 5.4 mg chlorothalonil and 0.09 mg chlorpyrifos (“C+C” group), with each pesticide group dissolved in 100 mL of acetone. Full and partial foundation frames were sprayed on both sides with a total of 10 mL or 5 mL of solution, respectively.

### 2.3. Colony Growth Measurements

We followed the growth of new colonies over time by measuring a number of variables from 18 May (day 0) to 17 October 2017 (day 146 after colony establishment). These included the total combined amount of newly constructed worker and drone comb, the total combined amount of sealed worker and drone brood produced, the total combined amount of honey, nectar, and pollen stored (food storage), and the estimated adult bee population. We used a gridded, wooden frame that consisted of 136 separate 1 in ×1 in squares to count the total area occupied by each variable on both sides of each frame, as described previously [16,21]. We also counted the number of adult bees in 20 uniformly spaced 1 in ×1 in squares on both sides of each frame and used this to estimate the total adult population size of each colony, as done previously [16,21]. All variables were measured every three weeks for a total of seven sampling periods after the establishment date except for adult population size, which was estimated every six weeks for a total of four sampling periods. Additional frames were sprayed and added throughout the experiment to any hive in which comb production extended to all existing frames to ensure that each colony always had space to expand.

In addition, we performed *Varroa* mite counts on surviving colonies in September 2017 and again in May 2018 using the standard powder sugar shake method [22,23]. Briefly, one half cup of bees (approximately 300 individuals) were collected from the brood nest area and placed in a mason jar with a lid consisting of 2 mm × 2 mm hardware cloth. Two tablespoons of powder sugar were added through the mesh and the jar was shaken for 60 s to coat the bees with sugar. The jar was flipped upside down and shaken for 60 s over a white surface to collect fallen phoretic mites, which were counted and extrapolated to the approximate number of phoretic mites per 100 bees. All colonies were treated for *Varroa* infestation with vaporized oxalic acid at the legal label dose rate in November 2017 and were not treated again until after the overwintering survival assessment was made the following year.

Colony survivorship was recorded every three weeks from May to October 2017 and again in May 2018 (one year after colony establishment) for a total of eight sampling periods. A colony was removed from the study if it was deemed dead or absconded on any given sampling day.

### 2.4. Statistical Analyses

To test the effects of pesticide residues in the wax-coated foundation on colony growth, we performed a repeated measures analysis of variance (ANOVA) test for each colony growth parameter [24]. Because the measurements of growth were taken from the same colonies over time, the model was built to test the main effects on colony growth of the treatment group, the sampling day, and their interaction. For all variables measured, the interaction effects of treatment group and sampling day were not significantly different. Therefore, we did not conduct pair-wise tests of mean values for any colony growth variable [24].

To test the effects of pesticide treatment on overall colony survivorship, we conducted a non-parametric Kaplan–Meier survival analysis [25]. *Varroa* infestation rates and winter survivorship were analyzed using *t*-tests through the standard least squares model platform. All tests were performed using the statistical software JMP^®^ 12.0 (SAS Inc., Cary, NC, USA). We set the level of statistical significance at α = 0.05 for all tests and reported all descriptive statistics as means ± standard errors of the mean (S.E.M.).

## 3. Results

**Figure 1:**
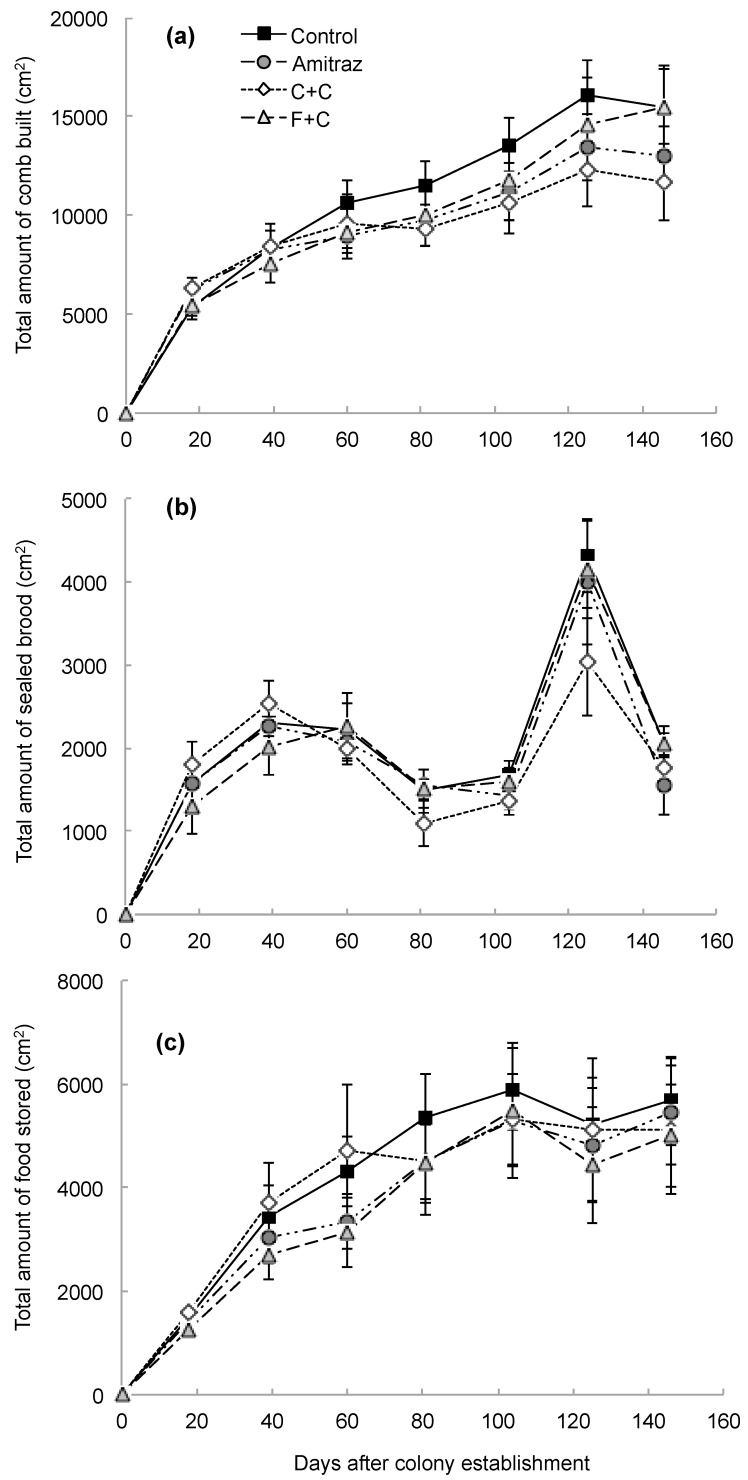
Growth patterns (panels **a**–**c**) for new honey bee colonies established on 18 May 2017 (day 0). Of the 30 total colonies, seven were established on wax foundation treated with amitraz, seven with chlorothalonil and chlorpyrifos (“C+C” treatment), seven with tau-fluvalinate and coumaphos (“F+C” treatment), and nine served as controls that were established on pesticide-free wax foundation. Colonies were sampled every three weeks through 17 October 2017 (day 146) for a total of seven sampling periods. Data are presented as the mean ± S.E.M.

We did not find any significant differences in growth between colonies established on pesticide-free foundation and those established on pesticide-laden foundation. All 30 of the newly established colonies grew steadily and in a similar pattern throughout the sampling period, having built an overall average 13,907 ± 1872 cm^2^ of combined worker and drone comb by 17 October, the last day we measured colony growth. There were no differences in growth between any of the treatment groups and the control group (F_3,21_ = 0.49, *p* = 0.69; Figure 1a).

**Figure 2:**
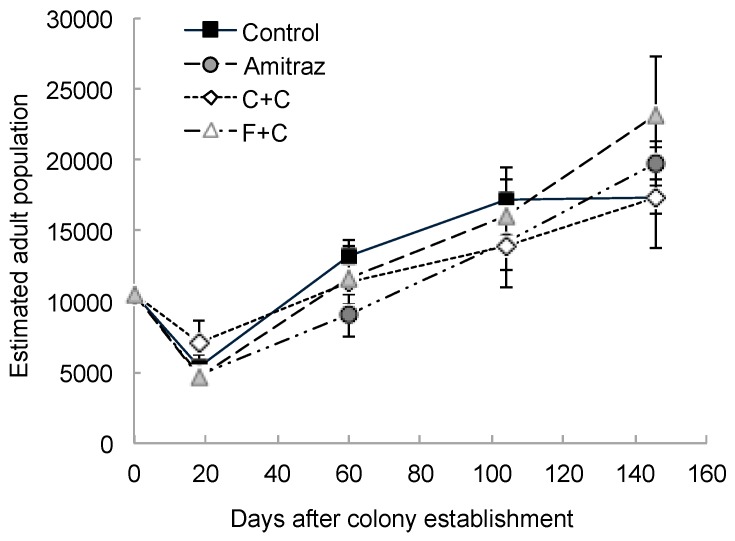
Estimated adult population for each of the 30 new honey bee colonies established on 18 May 2017 (day 0). There were seven colonies in the amitraz treatment group, seven in the chlorothalonil and chlorpyrifos treatment group (“C+C”), seven in the *tau*-fluvalinate and coumaphos treatment group (“F+C”), and nine in the control group. Each colony was started with an artificial swarm containing 3 lbs of adult bees, or approximately 10,476 individuals [18]. Population size was estimated every six weeks through 17 October 2017 (day 146) for a total of four sampling periods. Data are presented as the mean ± S.E.M.

A similar pattern of brood production was observed in all colonies, with the highest overall average of brood produced being 3881 ± 611 cm^2^ in September. No differences between the treatment groups and the control group were observed, however (F_3,20_ = 0.34, *p* = 0.80; Figure 1b). Food storage was approximately even across colonies, with colonies having stored an average of 5320 ± 1032 cm^2^ of honey, nectar, and pollen by the last day of data collection. There were no differences in food storage between the treatment groups and the control group (F_3,22_ = 0.28, *p* = 0.84; Figure 1c). We observed a drop in the adult population size estimates 18 days after colony establishment, which was likely caused by the bees from the original packages dying off and the first batches of new brood not having emerged yet. After this dip, there was a steady and similar pattern of population growth for all colonies, reaching an overall average of 19,410 ± 2611 adult bees per colony by the last sampling day in October. Similar to the previous measures of colony growth, there was no difference in population size between any of the treatment groups and the control group (F_3,21_ = 0.34, *p* = 0.79; Figure 2). There was a significant effect of the sampling day on all measures of colony growth (*p* < 0.0001), but there was no interaction effect between the treatment groups and the sampling day (see Table 1 for all statistical values).

The experiment began with seven colonies in each of the pesticide treatment groups and nine colonies in the control group, for a total of 30 colonies. Interestingly, in the time period between colony establishment (day 0) and the first sampling period (day 18), two of the seven amitraz colonies, two of the seven C+C colonies, and one of the seven F+C colonies had died prior to any data collection. None of the control colonies died during this time period.

**Figure 3:**
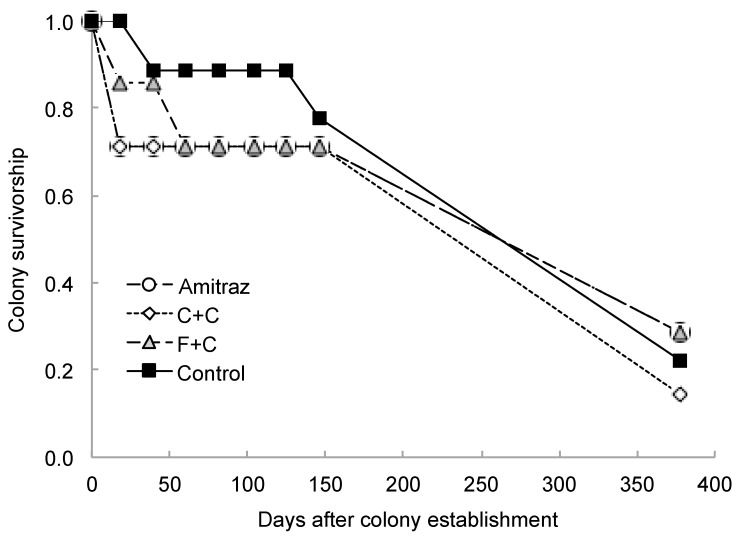
Survivorship curves for colonies established using frames with plastic foundation coated with pesticide-free wax (Control group) or wax exposed to either amitraz (“amitraz” group), a mix of chlorothalonil and chlorpyrifos (“C+C” group), or a mix of *tau*-fluvalinate and coumaphos (“F+C” group). Colony survival was evaluated every three weeks from May to October 2017. A final evaluation of survivorship was done one year from the establishment date on May 2018. A colony was removed from the study if it was deemed dead or absconded on a given sampling day.

This survivorship trend may merit further study, although no summer die off was significantly different between treatment groups. By the last sampling period (day 125), five of the initial seven colonies for all treatment groups and seven of the nine control colonies were still alive. Overwintering survival was low across all treatments, with only seven of the initial 30 colonies remaining alive by 18 May 2018 (day 378). The surviving group consisted of two colonies in the amitraz group, one colony in the C+C group, two colonies in the F+C group, and two colonies in the control group (Figure 3). We found no differences in overwintering survival between colonies in the control group and those in any of the pesticide treatment groups (χ^2^ = 1.15, *p* = 0.76; Figure 3).

**Figure 4:**
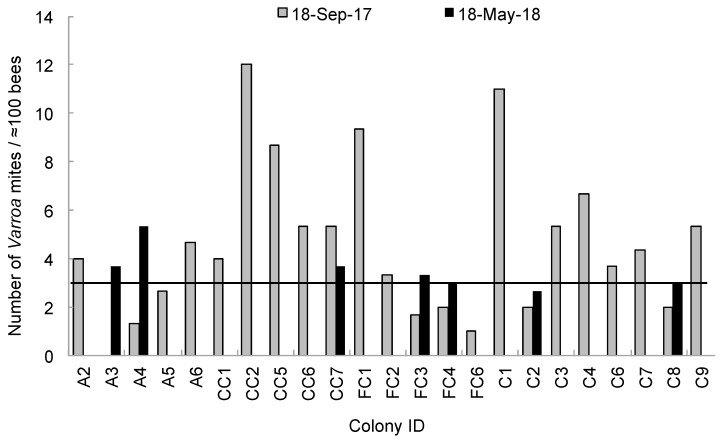
Estimated number of *Varroa destructor* mites per approximately 100 bees (primary y-axis) obtained from experimental colonies using the powder sugar shake method. The x-axis denotes each colony and the experimental group it belonged to. Experimental treatments include colonies in the amitraz group (A), the chlorothalonil and chlorpyrifos mix group (CC), the *tau*-fluvalinate and coumaphos mix group (FC) and the control group (C). The first *Varroa* mite count was done on 18 September 2017 (grey bars) and the second count was done on 18 May 2018 (black bars) for all colonies that were alive on that date. The horizontal black line indicates the threshold of three mites per approximately 100 bees (i.e., 3% infestation level) above which it is typically recommended to perform some type of *Varroa* control.

With respect to *Varroa* mite infestation levels, most of the colonies that had a phoretic mite infestation above 3% in September 2017 (65.2%) had died by May 2018. The colonies that died had significantly more *Varroa* (mean = 5.71 mites/≈100 bees) than those that had survived (mean = 2.05 mites/≈100 bees) by May 2018 (*t*-ratio = 2.96; *p* = 0.0075), independent of the treatment group. Six of the seven colonies that survived the full year of study (85.7%) had a phoretic mite level at or below 3% in September 2017, with the exception of one C+C colony that had 5.33 mites per ≈ 100 bees (Figure 4).

## 4. Discussion

We sought to discover the effects of initial pesticide contamination in wax foundation on the growth and survival of newly established honey bee colonies. Our results showed that initial contamination of beeswax foundation with field-relevant concentrations of amitraz alone, a combination of *tau*-fluvalinate and coumaphos, or a combination of chlorpyrifos and chlorothalonil at field-relevant concentrations did not have significant effects on colony growth or overwintering survival.

Initially, the neutral response of incipient colonies to pesticide-laden wax foundation could be considered positive news for the beekeeping industry, given that the many studies showing negative effects of pesticide contamination on bee health at the individual level have elevated concerns about pollinator–pesticide interactions in the brood rearing environment. That said, it seems potentially disastrous to take these results at face value and conclude definitively that pesticide exposure of bees through the beeswax appears to have no impact on colony health or productivity. A hive’s beeswax matrix is highly lipophilic, so the active ingredients of pesticides quickly enter and stay in this matrix years after a product is no longer used [12]. For instance, recent studies continue to show that beekeeper-applied miticides are frequently found in wax [11,26], which is a significant cause for concern, given that all stages of bee development, in addition to food storage, occur in conjunction with the beeswax matrix. This leads to constant and varying levels of pesticide exposure to all colony members over time [27], especially when the same comb is used for multiple seasons without being replaced. For instance, pesticide application to adult workers of varying ages and genotypes has been shown to cause synergistic and negative effects on the survival of larvae and adults and on the production of cytochrome P450 detoxification enzymes [28,29,30,31,32].

Furthermore, the presence of amitraz, *tau*-fluvalinate, and coumaphos in the brood-rearing wax environment has been shown to cause lower spermatozoa viability in queen spermathecae, lower queen reproductive potential, higher supersedure rates, and lower egg-laying rates [16,17,33,34,35,36,37]. These miticides also affect drone development, as they can reduce drone production [38] and survival [39], as well as spermatozoa production and viability [40,41,42,43].

Like other organophosphates, chlorpyrifos inhibits the production of acetylcholinesterase, which prevents the processing of neurotransmitters at nerve synapses [44]. Although not much is known about the effects of chlorpyrifos on honey bees, it can cause substantial synergistic effects when combined with other pesticides, leading to high larval mortality [45]. Moreover, a recent study in which wax was contaminated with field-relevant concentrations of chlorpyrifos in combination with chlorothalonil showed decreased spermatozoa viability in sexually mature drones [43]. The most recent data published has estimated that, conservatively, the U.S. used over 5 million pounds of products with chlorpyrifos as the active ingredient from 2000–2010. This is a decline in chlorpyrifos use, as more than 10 million pounds per year was used in the 1990’s [8].

Chlorothalonil is a non-systemic fungicide known to reduce intracellular levels of the antioxidant molecule glutathione, causing poor enzymatic activity and, eventually, fungal cell death [46]. Chlorothalonil was shown to cause high larval mortality when larvae were fed bee bread contaminated with this fungicide [32]. The rate of mortality was even higher when it was mixed at high concentrations with *tau*-fluvalinate, coumaphos, or chlorpyrifos, although this was not true at more diluted concentrations of the fungicide [32]. Chlorothalonil is also considered a “marker” that activates honey bee pollen-entombing behaviors [9,47]. Entombing is suspected to be a protective behavior consisting of storing pollen in sunken or capped wax cells, and it is associated with high colony mortality. It is estimated that over 8 million pounds of products with chlorothalonil as the active ingredient have been used in U.S. agriculture every year since 1992, with use reaching over 11 million pounds in 1997. These estimates do not account for non-agricultural use, which would increase its use to approximately 15 million pounds per year since the 1990’s [8]. Despite their potential harm to colony health, both chlorothalonil and chlorpyrifos are licensed for use on honey bee-pollinated crops, which explains their high prevalence in honey bee colonies [11,12].

Our study did not find a significant effect of any pesticide treatment group on *Varroa* mite levels. This is not surprising in regards to *tau*-fluvalinate and coumaphos, as both are ineffective against *Varroa* [48,49,50,51,52]. However, it is intriguing that amitraz-coated foundation did not cause lower *Varroa* levels in our study, given that it is still an effective treatment for *Varroa* control in the U.S., and mite resistance to this product has only been reported in a few regions within North America [9,51]. When comparing the level of *Varroa* taken in September 2017 with the survivorship of colonies in May 2018, colonies with mite loads above the threshold of three mites per 100 bees [6] had a significantly higher chance of death after the overwintering period compared to colonies that were below this mite threshold. Our results are thus congruent with the notion that high *Varroa* mite infestations, and likely high *Varroa*-vectored virus loads, continue to be the main culprit of colony mortality [6,53,54]. It also reinforces the recommendation that beekeepers should perform some type of *Varroa* control in late summer or early fall when their colonies exhibit a phoretic mite level above the 3% threshold, and they should be aware of colonies with high mite infestation that could potentially cause drift of phoretic mites to other nearby colonies.

In the future, similar studies should track other variables of colony health. For instance, measuring the production of propolis, which is known as a way for colonies to “self-medicate” against pathogens [55,56], could yield interesting information on how colonies deal with pesticide exposure. Other variables could include measuring brood production patterns and how they relate to queen quality [16,57], the identification of pollen entombing behavior, measuring *Varroa*-vectored virus levels, measuring the creation of burr comb in uncontaminated areas of the hive, or determining other routes of pesticide exposure in the hive [47,58]. For instance, it is possible that our colonies were further exposed to additional pesticides via foraging activities. However, this route of pesticide exposure was likely minimal due to the apiary location and the absence of any entombed pollen, which seems to indicate that pesticide contamination of pollen, at least with chlorothalonil, was probably low [47]. Furthermore, the area surrounding our apiary site was predominantly cattle pastures as opposed to commercial cropland, so there was likely few pesticides applied to agroecosystems around our apiary. Interestingly, although it was cost prohibitive to conduct pesticide residue analysis on our comb samples, we noticed a large amount of burr comb constructed throughout the course of the study, which could have been done as an attempt by the bees to avoid the pesticide-laden foundation, although pesticides can move down the concentration gradient in beeswax to contaminate previously clean beeswax [59]. Another study showed that pesticide contaminated beeswax had sub-lethal effects on worker bees by causing a premature shift in temporal polyethism [59]. If this happened in our colonies, it did not cause noticeable effects on a population level. While none of these variables would explain colony health status as elegantly as the estimates of colony growth and overwintering survival would, they could potentially shed further light on how new colonies react to contaminated comb.

## 5. Conclusions

Ultimately, it would be irresponsible to use our results to conclude that initial contamination of wax foundation with the five pesticides we examined has no impact on colony health. However, it is possible to conclude that new colonies are indeed able to flourish in a pesticide-laden wax environment if these pesticides occur at field-relevant concentrations and no additional pesticides are introduced into the hive. Furthermore, our results indicate that high *Varroa* infestation levels—and potentially high virus levels—are a recipe for colony demise.

Studies on the toxicological effects of pesticides on honey bees are commonly done using individual bees and unrealistic pesticide exposure routes or dosages. To date, there still exists a literature gap in our knowledge about the synergistic effects of pesticide contamination at the colony level using realistic routes of exposure and field-relevant concentrations of the products tested. While the economic and temporal costs of conducting such colony-level assessments are high, the benefits of obtaining these data will increase our understanding of how pesticide exposure affects overall honey bee health.

## Figures and Tables

**Table 1:** Statistical values for repeated measures analysis of variance (ANOVA) tests performed for each of the four variables of colony growth assessed from new honey bee colonies established in 2017. There was a significant effect of sampling day for all variables. However, there was neither a significant effect of pesticide treatment group (i.e., amitraz alone, a mix of chlorothalonil and chlorpyrifos, or a mix of *tau*-fluvalinate and coumaphos), nor an interaction effect between pesticide treatment group and sampling day on any of the variables measured. Significant statistical values are shown in bold.

Parameter Measured	Effect of Pesticide Treatment				Effect of Sampling Day				Interaction Effect			
	DF_nu_	DF_den._	F-Value	*p*-Value	DF_nu_	DF_den._	F-Value	*p*-Value	DF_nu_	DF_den._	F-Value	*p*-Value
Amount of comb built	3	21	0.49	0.69	7	136	131.18	**<0.0001**	21	136	1.36	0.15
Amount of brood produced	3	20	0.34	0.80	7	136	68.00	**<0.0001**	21	136	0.88	0.61
Amount of food stored	3	21	0.28	0.84	7	136	55.70	**<0.0001**	21	136	0.34	1.00
Estimated adult population	3	21	0.34	0.79	4	79	48.77	**<0.0001**	12	79	1.29	0.24
